# Securing IoT Vision Systems: An Unsupervised Framework for Adversarial Example Detection Integrating Spatial Prototypes and Multidimensional Statistics

**DOI:** 10.3390/s25216658

**Published:** 2025-11-01

**Authors:** Naile Wang, Jian Li, Chunhui Zhang, Dejun Zhang

**Affiliations:** School of Cyberspace Security, Beijing University of Post and Telecommunications, Beijing 100876, China; wangnaile@bupt.edu.cn (N.W.); zhangchunhui@bupt.edu.cn (C.Z.); zhangdejun@bupt.edu.cn (D.Z.)

**Keywords:** adversarial example detection, IoT security, unsupervised detection, spatial statistics, Mahalanobis distance

## Abstract

The deployment of deep learning models in Internet of Things (IoT) systems is increasingly threatened by adversarial attacks. To address the challenge of effectively detecting adversarial examples generated by Generative Adversarial Networks (AdvGANs), this paper proposes an unsupervised detection method that integrates spatial statistical features and multidimensional distribution characteristics. First, a collection of adversarial examples under four different attack intensities was constructed on the CIFAR-10 dataset. Then, based on the VGG16 and ResNet50 classification models, a dual-module collaborative architecture was designed: Module A extracted spatial statistics from convolutional layers and constructed category prototypes to calculate similarity, while Module B extracted multidimensional statistical features and characterized distribution anomalies using the Mahalanobis distance. Experimental results showed that the proposed method achieved a maximum AUROC of 0.9937 for detecting AdvGAN attacks on ResNet50 and 0.9753 on VGG16. Furthermore, it achieved AUROC scores exceeding 0.95 against traditional attacks such as FGSM and PGD, demonstrating its cross-attack generalization capability. Cross-dataset evaluation on Fashion-MNIST confirms its robust generalization across data domains. This study presents an effective solution for unsupervised adversarial example detection, without requiring adversarial samples for training, making it suitable for a wide range of attack scenarios. These findings highlight the potential of the proposed method for enhancing the robustness of IoT systems in security-critical applications.

## 1. Introduction

The rapid proliferation of Internet of Things (IoT) systems, such as smart surveillance, intelligent transportation, and medical IoT, has greatly benefited from recent advances in deep learning. Convolutional neural networks (CNNs) and other deep architectures have achieved remarkable success in visual recognition tasks, enabling IoT devices to perform real-time perception and decision-making. However, despite their success, deep learning models have been shown to be highly vulnerable to adversarial examples [1]. By adding perturbations that are imperceptible to the human eye, adversarial examples can mislead models into making incorrect predictions, thereby posing a serious threat to the deployment of deep learning in safety-critical IoT scenarios such as autonomous driving and smart healthcare.

To address this issue, researchers have developed various adversarial attack methods and corresponding defense or detection techniques. Much of the existing studies focus on gradient-based optimization attacks, such as the fast gradient sign method (FGSM) [2], the basic iterative method (BIM) [3], and projected gradient descent (PGD) [4], which achieve effective attacks by iteratively updating the perturbation direction. Optimization-based methods like C&W [5] and DeepFool [6] further enhance attack efficacy by formulating objective functions to find perturbations near the decision boundary. More recently, stronger and more transferable attacks, particularly GAN-based adversarial generators, have gained attention; for instance, the Generative Adversarial Network (AdvGAN) [7] and its subsequent variants demonstrate that GAN-based attacks can generate high-quality, efficiently produced adversarial examples that are more stealthy and easily transferable across models than many optimization-based attacks [8]. These advances have driven the development of adversarial detection and defense research.

Current detection approaches often rely on model confidence scores, energy functions, or statistical modeling in the feature space, and have shown promising results against common attacks such as PGD and C&W. However, these methods generally assume access to labeled adversarial data or depend on a single detection perspective, which limits their adaptability to new generative attacks like AdvGAN. In practical IoT scenarios, where devices operate with limited computational resources and unknown attack types, such dependence on prior adversarial knowledge greatly reduces usability.

Recent quantitative studies highlight the efficiency and robustness of spatial prototype-based and multidimensional statistics-based anomaly detection frameworks, motivating their potential application in IoT adversarial example detection. For instance, PaDiM and its derivatives [9], which construct multivariate Gaussian statistics over spatial feature embeddings, achieved mean AUROC scores exceeding 97% on MVTec AD and 95% on VisA, demonstrating that prototype-style spatial modeling can effectively capture local deviations in high-dimensional convolutional feature maps. Similarly, PatchCore [10] and ProtoAD [11] show that integrating spatially localized prototypes with multivariate statistical estimation not only improves detection accuracy but also reduces inference time by 30–40% compared to dense pixel-level approaches. In parallel, Mahalanobis distance-based multidimensional modeling has proven effective in out-of-distribution (OOD) and adversarial contexts: Lee et al. [12] report 10–15% AUROC gains in adversarial detection benchmarks when incorporating class-conditional feature distributions. These results quantitatively demonstrate that both spatial prototype statistics and multidimensional feature modeling provide measurable gains in detecting abnormal inputs.

Nevertheless, important gaps remain. While spatial prototypes and Mahalanobis-based detectors have shown promise, their integration in unsupervised, IoT-oriented adversarial detection remains under-explored [9,13]. Addressing this gap is critical for resource-constrained deployments where collecting labeled adversarial samples is impractical and computational resources are limited.

To bridge this gap, this study proposes an unsupervised adversarial detection framework that integrates spatial statistical prototypes and multidimensional feature distribution modeling. Unlike conventional detectors, it requires only clean samples for training and captures both semantic-level deviations and global distributional shifts, thereby improving robustness against unseen generative attacks. The proposed framework is implemented on the CIFAR-10 dataset [14] with AdvGAN-based adversarial samples generated at four attack intensities. Two baseline architectures, VGG16 and ResNet50, are adopted to validate the framework, and its effectiveness is evaluated through comparative and ablation experiments.

To comprehensively validate the method, ablation studies are conducted to assess the individual and combined effects of each detection module. Five unsupervised detection baselines, U-CAN [15], feature-space neighborhood detection (Deep kNN) [16], feature squeezing (FS) [17], Magnet [18] and Lightweight Bayesian Refinement (LiBRe) [19], are introduced for comparison. Experiments are evaluated using AUROC and AUPR metrics and further extended to traditional attacks including FGSM, PGD, and C&W. Moreover, cross-dataset experiments on Fashion-MNIST [20] are conducted to validate generalization across data domains. Results demonstrate that the proposed unsupervised framework effectively detects AdvGAN and traditional attacks with high accuracy and strong cross-dataset robustness, showing practical potential for IoT security.

The main contributions of this paper are threefold:

(1) A novel unsupervised framework that integrates spatial prototype similarity and Mahalanobis-based multidimensional analysis for detecting generative adversarial attacks, such as AdvGAN, in IoT vision systems, requiring no adversarial samples for training.

(2) Demonstrated versatility and robustness through extensive evaluation against both generative (AdvGAN) and traditional attacks (FGSM, PGD, C&W), achieving high detection performance (AUROC > 0.95) across different models and datasets.

(3) Validation of practical applicability for resource-constrained IoT deployments, evidenced by the method’s lightweight design, computational efficiency, and strong cross-dataset generalization on CIFAR-10 and Fashion-MNIST.

The remainder of this paper is organized as follows. Section 2 reviews related work on adversarial attack methods and adversarial example detection methods. Section 3 presents our methodology, including adversarial example generation, baseline model training, detection modules, and fusion strategy. Section 4 details experimental design, comparative experiments, ablation studies, traditional attacks, and cross-dataset generalization experiments. Section 5 concludes the paper.

## 2. Related Work

### 2.1. Adversarial Attack Methods

Traditional adversarial attacks, such as FGSM, PGD, and C&W, are typically based on gradient computation or iterative optimization to generate adversarial examples, often aiming to minimize perturbation norms. However, these approaches exhibit certain limitations in terms of efficiency and perceptual quality. For instance, FGSM is simple and efficient, involving only a single gradient update, which results in low computational overhead. Nevertheless, its attack strength is limited, and it can be easily defended against. PGD improves attack success rates through multi-step iterations and random initialization, but incurs higher computational costs and may still become trapped in local optima, leading to attack failure against some highly defensive models. C&W is capable of generating strong adversarial examples; however, its optimization process is complex and computationally expensive, making it less practical for large-scale datasets or real-time applications.

In contrast, the generative attack method AdvGAN introduces a generative adversarial network to learn the distribution of adversarial perturbations. Once training is completed, it can rapidly generate perturbations for arbitrary inputs without per-sample optimization. Its adversarial training mechanism ensures that perturbations remain consistent with the original data in perceptual quality, making them harder to detect, while also supporting semi-white-box and black-box attacks. Through distilled training with substitute models, AdvGAN can also achieve high attack success rates. Experimental results have demonstrated that AdvGAN maintains success rates above 90% against defenses such as adversarial training on datasets like MNIST and CIFAR-10, while achieving extremely high generation efficiency (less than 0.01 s per sample). Despite its relatively high training cost and reliance on initial white-box access to train the generator, its efficiency, stealthiness, and practicality make it a representative generative attack method, particularly suitable for large-scale or real-time IoT applications.

In the context of IoT security, adversarial attacks pose severe risks to applications such as intelligent transportation, smart healthcare, and industrial IoT, where real-time decision-making is critical. Therefore, exploring efficient and transferable attack methods like AdvGAN is of particular importance for understanding IoT vulnerabilities.

### 2.2. Adversarial Example Detection Methods

Adversarial example detection is a key research direction in the field of deep learning security, aiming to identify malicious inputs that have been subtly perturbed to cause model misclassification. Existing detection approaches can generally be categorized into supervised, unsupervised, and feature space-based methods.

(1) Supervised and model-driven approaches: Classical detectors such as Feature Squeezing (FS) [17] and MagNet [18] rely on transformation and reconstruction techniques. FS reduces input resolution or color depth to reveal perturbation inconsistencies, while MagNet employs autoencoders to project inputs back onto the manifold of clean samples before classification. Although effective against simple gradient-based attacks, these methods depend on labeled adversarial data and perform poorly when facing unseen or generative attacks.
(2) Unsupervised and representation-based approaches: To overcome this limitation, unsupervised detection frameworks have been developed. The Deep k-Nearest Neighbors (Deep kNN) [16] method identifies adversarial examples by analyzing the consistency of feature-space neighbors across layers. The U-CAN method [15] constructs unsupervised activation clusters to identify samples that deviate from normal feature distributions, showing high adaptability to unknown attack types. Meanwhile, Lightweight Bayesian Refinement (LiBRe) [19] leverages Bayesian uncertainty estimation to refine decision boundaries without adversarial labels, achieving lightweight and efficient detection suitable for resource-constrained IoT devices.
(3) Statistical and frequency-based approaches: Some detection strategies analyze the structural and statistical properties of adversarial perturbations. The Expected Perturbation Score (EPS) [21] method evaluates prediction stability under controlled perturbations, while entropy-based [22] and frequency-domain [23] detectors assess entropy or spectral variations induced by adversarial noise. Graph-based methods, such as the Latent Neighborhood Graph (LNG) [24], convert feature relations into graph structures and employ attention mechanisms to detect anomalies.

Recent studies extend adversarial detection to IoT intrusion scenarios. Hybrid CNN–BiLSTM frameworks capture temporal–spatial correlations in IoT traffic data [25], while transformer-based detectors enhance accuracy and scalability [26]. GAN-based anomaly detection has also been applied in fog or edge environments [27], demonstrating robustness under limited computing resources. These developments highlight the growing demand for lightweight and unsupervised adversarial detection frameworks capable of real-time deployment in IoT systems.

### 2.3. Discussion and Research Motivation

Despite substantial progress, existing detectors still face challenges in handling generative adversarial attacks such as AdvGAN. Supervised detectors like FS [17] and MagNet [18] depend on labeled data and fail to generalize to novel attacks. Unsupervised frameworks such as Deep kNN [16] and U-CAN [15] focus primarily on feature-space similarity but overlook the fine-grained statistical deviations introduced by generative models. LiBRe [19], although efficient, lacks spatial interpretability and sensitivity to localized perturbations.

To address these limitations, this study proposes an unsupervised adversarial example detection framework integrating spatial prototype statistics and multidimensional feature consistency analysis. The proposed method models the intrinsic distribution of clean data and detects adversarial anomalies through statistical deviations in both spatial and multidimensional feature spaces. This design not only aligns with realistic IoT threat models, where labeled attacks are unavailable, but also bridges the gap between spatial interpretability and statistical robustness, providing a practical and theoretically grounded solution for IoT adversarial detection.

To further clarify the novelty and positioning of the proposed method, Table 1 summarizes representative recent state-of-the-art adversarial detection methods (2018–2025). For fair comparison, the reported results of prior methods and our own experiments were all obtained on the CIFAR-10 dataset, which serves as a widely used benchmark for adversarial detection studies. As shown, most existing approaches rely on labeled data, additional training, or single-perspective modeling. In contrast, our proposed unsupervised dual-module framework simultaneously captures semantic-level spatial consistency and global statistical anomalies, achieving superior robustness and efficiency suitable for IoT deployment.

This table highlights that, unlike previous single-perspective or supervised detectors, the proposed approach requires no adversarial training samples, combines spatial interpretability with statistical sensitivity, and achieves state-of-the-art performance under both traditional and generative attacks, offering a balanced solution for real-world IoT security applications. Table 1 mainly summarizes the methodological aspects of recent state-of-the-art detectors, whereas the quantitative performance results under unified experimental settings are provided and discussed in detail in Section 4.2 (Comparative Experiments).

## 3. Methodology Design

### 3.1. Method Overview

In the context of IoT vision systems, the adversary aims to generate visually indistinguishable yet malicious inputs that can mislead the target model’s predictions. Specifically, this work focuses on generative adversarial attacks, where a generator, such as AdvGAN, learns to produce adversarial examples through an optimization process guided by the target model.

The adversary is assumed to have white-box access during the generator training stage, allowing gradient-based optimization to synthesize adversarial perturbations. During inference or deployment, however, the defender (our detection framework) operates in a black-box setting, relying only on clean data and extracted features without access to model gradients or adversarial labels. The attacker seeks to cause misclassification while maintaining perceptual similarity to the original input.

The proposed detector assumes no prior knowledge of the attack type or samples. It utilizes clean samples to model the intrinsic data distribution and detect distributional deviations caused by generative adversarial perturbations. This setting reflects realistic IoT scenarios, where obtaining adversarial examples in advance is often infeasible, and detection must be both unsupervised and lightweight.

**Figure 1:**
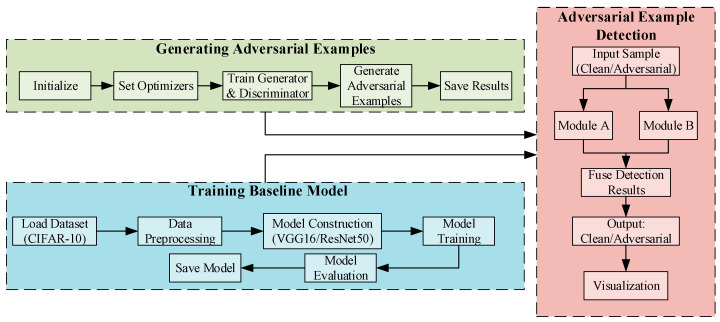
Overall framework of the proposed method.

The overall framework of the proposed detection method is illustrated in Figure 1. First, adversarial examples with varying perturbation intensities are generated using the AdvGAN attack. Subsequently, two deep convolutional networks, VGG16 and ResNet50, are trained as baseline classifiers and feature extractors. On this basis, two detection modules are introduced: (A) a prototype similarity-based detection method leveraging spatial statistics, and (B) an anomaly detection method based on multidimensional statistical features and Mahalanobis distance. Finally, Modules A and B are integrated to effectively distinguish clean samples from adversarial ones.

The design philosophy of this framework is rooted in addressing the fundamental limitations of existing approaches. Unlike detection methods that rely solely on the model’s output confidence (which can be misleadingly high in generative attacks like AdvGAN) or instance-level neighborhood analysis (for example, Deep kNN, which may suffer from the “curse of dimensionality” in deep feature spaces), our method probes the intrinsic statistical properties of the model’s internal representations. This offers a more robust and generalized signal for anomaly detection.

Theoretically, Module A operates on the principle of intra-class feature consistency. It posits that clean samples of a class will cluster around a prototypical representation in the spatial statistical feature space, while adversarial perturbations will cause a deviation. This provides a semantically meaningful measure of abnormality. In contrast, Module B is grounded in the theory of multivariate outlier detection. By capturing higher-order statistical moments and global correlations through the Mahalanobis distance, it sensitively identifies distributional shifts that may not be apparent in the spatial domain alone. The architectural innovation lies in the synergistic fusion of these two complementary perspectives: Module A ensures discriminability based on semantic coherence, while Module B enhances sensitivity to subtle, global distortions.

This dual-module collaborative architecture is fundamentally different from and theoretically superior to single-perspective methods. It effectively mitigates the risk of a single detection mechanism being circumvented, thereby providing a more comprehensive defense, particularly against sophisticated and stealthy generative attacks.

This design ensures that the proposed framework is lightweight and adaptable, making it suitable for IoT vision systems, where adversarial robustness is critical for secure deployment.

### 3.2. Adversarial Example Generation

**Figure 2:**
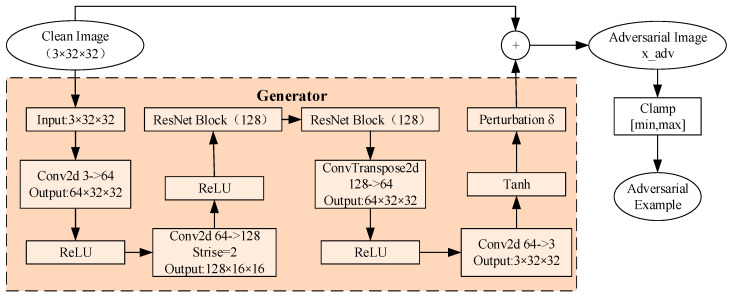
Process of generating adversarial samples.

In this study, the CIFAR-10 dataset is adopted as the experimental benchmark. This dataset contains 60,000 32 × 32 pixels color images across 10 categories, with 50,000 images used for training and 10,000 for testing. To construct a comprehensive adversarial detection platform, adversarial examples are first generated based on the AdvGAN framework. The generator architecture and the detailed process of generating adversarial examples are illustrated in Figure 2. By training the generator and discriminator and adjusting the upper limit *ε* of the perturbation magnitude, four sets of adversarial examples with different attack intensities are constructed (*ε* ∈ {0.05, 0.1, 0.2, 0.3}). The adversarial example is formulated as shown in Equation (1):

(1) $$x_{adv}=clip(x+\delta ,-\varepsilon ,+\varepsilon )$$

where *x* denotes the original input image, a tensor of dimensions 32 × 32 × 3 for CIFAR-10 datasets, with pixel values normalized to the range [0, 1] through division by 255 during preprocessing to ensure consistent input scaling; *δ* represents the adversarial perturbation generated by the AdvGAN generator, a tensor of the same dimensions as *x*, with values constrained to the range [−*ε*, +*ε*] per pixel, where *ε* is a scalar perturbation upper bound (taking values from {0.05, 0.1, 0.2, 0.3}) that controls the maximum allowable perturbation magnitude in the L∞ norm; and $x_{adv}$ is the derived adversarial sample, formed by adding *δ* to *x* and clipping the result to [0, 1] to maintain perceptual quality and validity for downstream tasks. This preprocessing ensures that all variables are unitless and operate within standardized ranges for effective adversarial example generation.

### 3.3. Baseline Model Training

In this study, two baseline classifiers, VGG16 and ResNet50 adapted for CIFAR-10, were trained and validated using the clean (unaltered) training set. These two deep convolutional neural networks have been widely applied in image recognition tasks, known for their stable architecture and reliable performance. When trained on the CIFAR-10 dataset, they not only achieve high classification accuracy but also provide hierarchical feature representations for subsequent detection modules.

Considering the relatively small resolution of CIFAR-10 images, the first convolutional layer of ResNet50 (7 × 7, stride = 2) was replaced with a 3 × 3 convolution (stride = 1, padding = 1). Additionally, the initial max-pooling layer was removed to prevent premature downsampling. The VGG16 model retained its standard backbone structure, with only the output dimension of the final fully connected layer adjusted to 10. During training, common data augmentation techniques, including random horizontal flipping and random cropping, were applied, along with normalization of the input data to improve generalization.

The models were optimized using stochastic gradient descent (SGD) with momentum (momentum = 0.9, weight_decay = 5 × 10^−4^). The initial learning rate was set to 0.01, with a batch size of 128 and a total of 60 training epochs. Specifically for ResNet50, a CosineAnnealingLR learning rate scheduler (T_max = 60) was additionally employed. A 10% subset of the training set was used as a validation set for model selection and early stopping, while final classification and detection-related metrics were reported on the independent test set. The resulting high-performance baseline models served a dual purpose: firstly, to measure the success rate of the AdvGAN adversarial attacks, and secondly, to act as feature extractors. They provided the input representations for the subsequent Module A, which is based on spatial statistics from convolutional layers and category prototype similarity, and Module B, which utilizes multidimensional statistics and Mahalanobis distance. This setup enabled the unsupervised detection evaluation aimed at distinguishing clean samples from adversarial samples.

### 3.4. Module A: Unsupervised Detection Based on Spatial Prototype Similarity

The intermediate features learned by deep neural networks for image classification tasks typically exhibit significant intra-class consistency and inter-class discriminability. However, adversarial examples often introduce subtle distributional shifts in the feature space, causing them to deviate from normal patterns. To capture such fine-grained anomalies, this study proposes Module A, whose core idea is to construct representative prototype vectors for each class in the feature space and measure the similarity between a test sample and the corresponding class prototype to identify abnormality.

Specifically, this study selects the conv4_3 and conv5_3 layers of VGG16, as well as the layer3 and layer4 layers of ResNet50, as the feature extraction layers. For an input sample *x*, the forward propagation produces a feature tensor $f\in R^{C\times H\times W}$, where *C* denotes the number of channels (such as 512 for VGG16 conv4_3), and *H × W* is the spatial resolution (such as 16 × 16 for VGG16 conv4_3). The values in *f* are non-negative real numbers due to the ReLU activation function, with typical ranges depending on the layer and training dynamics, but often bounded between 0 and 10 for stable networks. No additional preprocessing is applied to *f* beyond the initial image normalization.

This feature tensor *f* is then mapped into a spatial statistical feature vector *s* as defined in Equation (2):

(2) $$s=[\mu_{1},\mu_{2},\ldots ,\mu_{C},\sigma_{1},\sigma_{2},\ldots ,\sigma_{C}]$$

where $\mu_{C}$ and $\sigma_{C}$ represent the mean and standard deviation of the *c*-th channel, respectively. Specifically, for each channel *c*, $\mu_{C}$ is computed as the mean of all values in that channel, and $\sigma_{C}$ is the standard deviation. This process compresses each image into a statistical feature vector *s* ∈ *R*^2*C*^. The values in *s* are real numbers; $\mu_{C}$ ranges from 0 to positive values (depending on feature magnitudes), while $\sigma_{C}$ is non-negative. The units are unitless, as they are statistical moments derived from feature activations. The computation of *s* involves no further preprocessing, but it relies on the precomputed feature tensor *f*.

During training, the predicted label of the classifier is used as a reference. For each class *c*, let the set of statistical features from its training samples be defined as $D_{c}=\{s_{1},\;s_{2},\;\ldots ,\;s_{\left|D_{c}\right|}\}$, where ${|D}_{c}|$ denotes the number of samples belonging to class *c*. The prototype vector of class *c* is then defined as the mean of all feature vectors in $D_{c}$, as shown in Equation (3):

(3) $$p_{c}=\frac{1}{\left|D_{c}\right|}\sum_{s_{i}\in D_{c}}s_{i}$$

**Figure 3:**
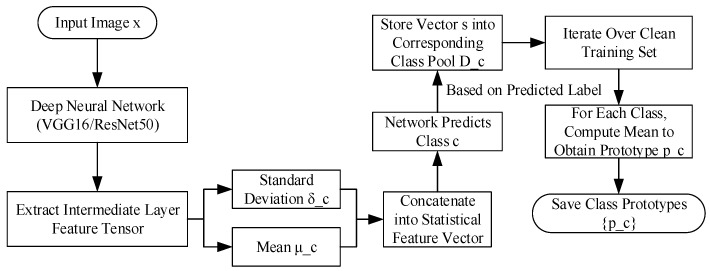
Module A class prototype computation process.

This yields a prototype representation $p_{c}$∈ *R*^2*C*^ for each class. The values in $p_{c}$ are derived from the means of the statistical features, so they inherit the same range and unitless nature as *s*. This prototype construction is performed offline during training, with no additional preprocessing steps. The overall training process is illustrated in Figure 3.

Module A’s design incorporates considerations for computational efficiency and theoretical robustness, making it suitable for resource-constrained IoT environments. The time complexity during feature extraction is O(C × H × W) for computing channel statistics. For example, with VGG16’s conv4_3 layer (*C* = 512, *H* × *W* = 16 × 16), this requires approximately 131,072 operations per sample. Prototype computation during training has O(N × 2C) complexity, but this is performed offline and does not affect inference time. During testing, the cosine similarity calculation requires only O(2C) operations per sample (such as 1024 operations for VGG16), ensuring real-time performance.

The space complexity is minimal, requiring storage of prototype vectors with O(K × 2C) complexity, where *K* is the number of classes. For CIFAR-10 (*K* = 10), this amounts to approximately 10 KB of memory, making it suitable for devices with limited resources.

In terms of bias-variance trade-off, Module A exhibits low variance due to the stable prototype-based approach but may introduce bias if prototypes cannot capture intra-class variations adequately. The fusion of multiple layers (such as conv4_3 and conv5_3) helps balance this trade-off by combining detailed spatial information with semantic representations.

The convergence properties are governed by the law of large numbers, with prototype estimates converging to their true values at a rate of O($1/\sqrt{N}$), where *N* is the number of training samples. This ensures stability even with moderate dataset sizes like CIFAR-10.

In the testing phase, the cosine similarity between the statistical feature vector s of a given sample and its corresponding class prototype $p_{c}$ is computed as follows:

(4) $$Sim(s,p_{c})=\frac{s{\cdot p}_{c}}{∥s∥∥p_{c}∥}$$

The cosine similarity ranges from −1 to 1, with values closer to 1 indicating higher similarity. A lower similarity score indicates a greater discrepancy between the sample and the normal class prototype, suggesting a higher probability of the sample being adversarial. To enhance robustness, the similarities from different layers are further weighted and fused according to Equation (5):

(5) $$Score(x)=\alpha \cdot {Sim}_{low}+(1-\alpha )\cdot {Sim}_{high},\alpha \in [0,1]$$

where *α* is the balancing coefficient, which is set to 0.5 in our experiments to equally weight the contributions from shallow and deep layers. ${Sim}_{low}$ is the similarity from a shallow layer (such as conv4_3), and ${Sim}_{high}$ is from a deep layer (such as conv5_3). The fused score, Score(*x*), ranges between −1 and 1, but in practice, it is used as an anomaly score where lower values indicate adversarial samples. The coefficient *α* is unitless and chosen empirically; however, our ablation studies show that the performance is robust to variations in α between 0.3 and 0.7.

This module effectively captures feature-space anomalies by leveraging prototype similarity, and the fusion strategy ensures stability across different attack intensities. All variables and features are derived through systematic steps, with clear ranges and units, ensuring reproducibility and transparency. The theoretical analysis demonstrates that Module A provides an efficient and robust solution for adversarial detection in IoT systems, with predictable computational characteristics and convergence properties.

### 3.5. Module B: Distribution-Based Anomaly Detection with Multidimensional Feature Fusion and Mahalanobis Distance

Adversarial examples not only alter low-order statistical properties of features, such as mean and variance, but also disrupt higher-order statistics (such as skewness and kurtosis) and global structural relationships (such as inter-channel correlations and singular value distributions). Relying solely on a single type of statistical measure (such as mean or variance) is insufficient to capture such complex anomalies. To address this limitation, this study proposes Module B, which constructs multidimensional statistical feature vectors and employs the Mahalanobis distance as an anomaly measure to more sensitively detect distributional deviations.

This study selects the conv4_3 and conv5_3 layers of VGG16, as well as the layer3 and layer4 layers of ResNet50, as the representation layers. For a given input sample, the feature tensor $f\in R^{C\times H\times W}$ is first extracted from these intermediate layers. This tensor is then flattened into a vector, and the following multidimensional statistical features are derived from it to form a comprehensive representation. The specific features extracted include:

(1) Global Mean and Variance: Scalar values computed across all elements of the flattened feature vector, capturing the overall magnitude and dispersion of the feature activations. The mean is a real number whose range depends on the layer’s activation distribution, while the variance is a non-negative real number.
(2) Skewness and Kurtosis: Scalar values that quantify the asymmetry and tailedness of the feature value distribution, respectively. These are unitless higher-order moments, with skewness being a real number that can be positive or negative, and kurtosis being a non-negative real number.
(3) Channel-wise Means and Standard Deviations: Vectors of length *C*, where each element is the mean or standard deviation computed from the activations of a single channel across its spatial dimensions (*H* × *W*). These features capture per-channel characteristics, with values being real numbers (means can be any real number, standard deviations are non-negative).
(4) First *k* Singular Values (SVD): A vector containing the largest *k* singular values (where *k* = 5 in this study) obtained from a truncated Singular Value Decomposition of the feature tensor *f* (reshaped into a matrix). These values are non-negative real numbers that reflect the dominant structural patterns in the feature space.

These features are concatenated into a unified statistical feature vector *f(x)*∈$R^{d^{\prime }}$, where *d*′ = 2 + 2 + 2*C* + *k*. For example, with *C* = 512 and *k* = 5, *d*′ = 1033. All components of *f(x)* are unitless, as they are derived from statistical moments of feature activations. No additional normalization is applied after concatenation; the values are used directly from the feature extraction process.

During training, a reference multivariate Gaussian distribution is established based on the statistical feature vectors *f*(*x*) from clean samples in the training set. The mean vector *μ*∈$R^{d^{\prime }}$ is computed as $\mu =\;\frac{1}{N}\sum_{i=1}^{N}f(x_{i})$, where *N* is the number of training samples. The covariance matrix Σ ∈ $R^{d^{\prime }\times d^{\prime }}$ is calculated as $\Sigma =\frac{1}{N}\sum_{i=1}^{N}(f(x_{i})-\mu )(f(x_{i})-\mu {)}^{⊤}$. To ensure numerical stability and invertibility, a small constant *ϵ* = 10^−6^ is added to the diagonal elements of Σ (regularization). This preprocessing step is critical to avoid singular matrices during inversion. The values in *μ* and Σ are unitless real numbers, with ranges dependent on the underlying statistical features.

In the testing phase, for any sample *x*, its statistical feature vector *f*(*x*)** is first computed. The Mahalanobis distance $D_{M}\left(x\right)$ is then evaluated using Equation (6):

(6) $$D_{M}\left(x\right)=\sqrt{\left(f(x)-\mu {)}^{⊤}\Sigma^{-1}(f(x)-\mu \right)}\;$$

**Figure 4:**
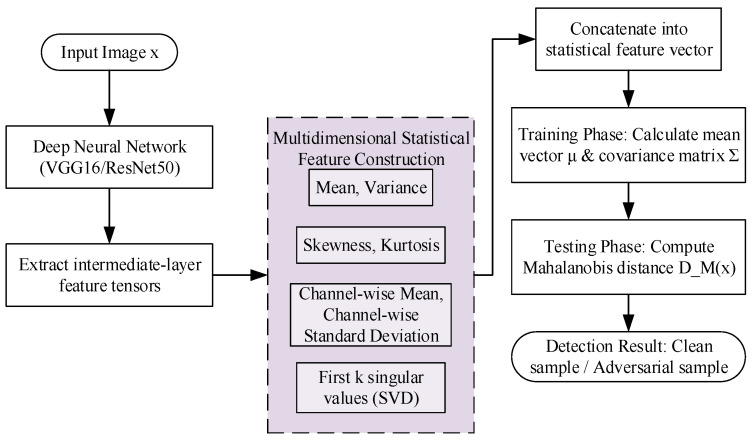
Module B detection process.

This distance quantifies the degree of deviation of the sample from the overall distribution. A larger $D_{M}\left(x\right)$ indicates a higher likelihood of the sample being an adversarial example. The entire detection process is illustrated in Figure 4.

Module B’s design incorporates multidimensional statistical features to capture complex distributional anomalies, with specific considerations for computational efficiency and theoretical robustness. The time complexity consists of three main components: feature extraction, statistical computation, and distance calculation. Feature extraction requires O(C × H × W) operations to flatten the tensor. Statistical computation involves O(C × H × W) for basic statistics (such as means and variances) and approximately O(k × C × H × W) for truncated SVD with k = 5. For VGG16 conv4_3 (*C* = 512, *H* × *W* = 256), this totals approximately 150,000 operations per sample. Mahalanobis distance calculation requires O(d’^3^) for covariance inversion (performed offline during training) and O(d’^2^) per sample during inference. With *d*′ = 1033, this amounts to approximately 10^6^ operations per sample, making it computationally feasible for batch processing but potentially intensive for real-time applications without optimization.

Space complexity is dominated by storing the mean vector *μ*(O(d’)) and covariance matrix Σ(O(d’^2^)). For *d*’ = 1033, this requires approximately 8 KB for *μ* (assuming double-precision floats) and 8 GB for Σ, which may be prohibitive for resource-constrained IoT devices. However, covariance approximation techniques (such as diagonalization or low-rank approximations) can reduce this requirement without significantly compromising detection accuracy.

In terms of bias-variance trade-off, Module B assumes a multivariate Gaussian distribution for the feature vectors, which may introduce bias if the true distribution deviates from this assumption (such as, in heavy-tailed scenarios). The regularization term *ϵ* = 10^−6^ reduces variance in covariance estimation, particularly important with limited training samples. The multidimensional feature approach mitigates bias by capturing higher-order statistics (such as skewness and kurtosis), but may increase variance due to the high-dimensional feature space. This trade-off is balanced through the Mahalanobis distance, which sensitively detects deviations while maintaining stability via regularization.

The Mahalanobis distance estimator exhibits convergence properties: the mean vector μ converges to the true mean at a rate of O($1/\sqrt{N}$) under the law of large numbers, and the covariance matrix Σ converges at a rate of O(1/*N*) for large *N*, assuming Gaussianity. The regularization ensures numerical stability, supporting consistent performance across dataset sizes. This analysis demonstrates that Module B provides a theoretically grounded and efficient approach for adversarial detection, with clear complexity characteristics that align with IoT constraints. The method’s robustness stems from its multidimensional feature fusion and regularization strategies, ensuring reliable performance in practical deployments.

### 3.6. Module Fusion: A Collaborative Detection Mechanism Integrating Spatial Statistics and Multidimensional Features

Although Module A and Module B can each capture feature deviations of adversarial examples at different levels, their focuses are distinct. Module A emphasizes the consistency of intra-class spatial statistics, detecting anomalies by measuring similarity to prototypes; Module B focuses on the integrity of global distributional characteristics and captures higher-order statistical differences using the Mahalanobis distance.

To fully exploit the complementary strengths of both methods and enhance the robustness and generalization capability of detection, this study integrates Module A and Module B. During the training phase, the scores from both Module A and Module B are calculated separately and standardized using the clean samples from the training set. For a given test sample, the raw scores obtained from the two modules are denoted as $score_{A}(x)$ and $score_{B}(x)$. These are then mapped into a normalized space using standardization, resulting in $sc\hat{o}re_{A}\left(x\right)$ and $sc\hat{o}re_{B}(x)$. Finally, a weighted sum is adopted to define the joint detection score:

(7) $$Score_{fusion}\left(x\right)=\alpha \cdot sc\hat{o}re_{A}\left(x\right)+(1-\alpha )\cdot sc\hat{o}re_{B}(x),\;\alpha \in [0,1]$$

where *α* is the balancing coefficient, set to 0.5 in the experiments.

**Figure 5:**
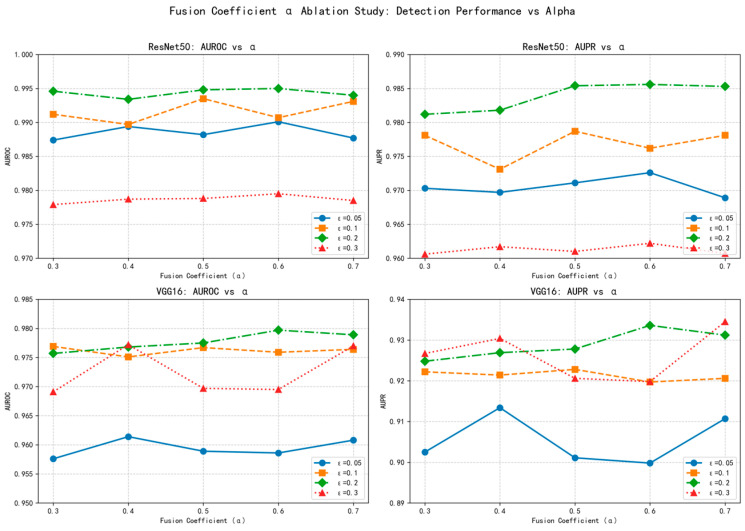
α Parameter robustness analysis across models and perturbation intensities.

As demonstrated in Figure 5, the fusion coefficient α exhibits remarkable robustness across a wide range of values (0.3 to 0.7). For both ResNet50 and VGG16 architectures, the detection performance (AUROC and AUPR) remains consistently high regardless of the specific α value chosen, with performance variations typically within 1–2% across the tested range. This stability is observed consistently across different perturbation intensities (*ε* = 0.05 to 0.3), confirming that the fusion mechanism is not sensitive to precise parameter tuning.

The observed robustness stems from the complementary nature of the two detection modules. Module A provides consistent intra-class discrimination through prototype similarity, while Module B offers sensitivity to distributional anomalies via Mahalanobis distance. Since both modules independently deliver strong detection signals, their weighted combination remains effective even with substantial variations in the fusion coefficient.

This parameter insensitivity is particularly advantageous for IoT applications, where automated deployment without manual fine-tuning is essential. The wide optimal range (*α* ∈ [0.3, 0.7]) ensures reliable performance without requiring precise parameter optimization, making our method practically deployable in resource-constrained environments.

In contrast, feature layer selection significantly impacts detection stability, as detailed in our ablation studies (Section 4.4). Our systematic evaluation shows that combining hierarchical features from different depths (conv4_3 and conv5_3 for VGG16; layer3 and layer4 for ResNet50) provides complementary advantages: shallower layers capture spatial details sensitive to subtle perturbations, while deeper layers encode semantic information robust to larger distortions. This multi-scale approach ensures comprehensive coverage of adversarial patterns across different perturbation intensities.

The motivation for this strategy lies in the fact that weighted summation is an intuitive and flexible linear fusion approach, capable of assigning interpretable contribution weights to both modules. The coefficient α balances the relative importance of class prototype matching (Module A) and global distributional consistency (Module B) in the final decision. The stability of α across different scenarios confirms that both modules provide strong, complementary detection signals, making the fusion robust to weight variations.

**Figure 6:**
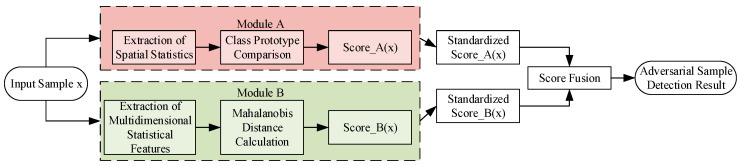
Integration of Module A and Module B.

This design allows the fused score to simultaneously account for semantic-level feature consistency and statistical-level distributional abnormality, thus forming a more comprehensive and stable decision boundary. As a result, the integrated mechanism significantly improves the generalization ability of detection against diverse adversarial attacks. The overall integration process is illustrated in Figure 6. By integrating the complementary strengths of the two modules, the fused detection mechanism offers a practical solution for IoT security applications, where reliable adversarial detection under resource constraints is particularly important.

## 4. Experimental Design and Analysis

### 4.1. Dataset and Adversarial Example Design

This study establishes the experimental platform based on the CIFAR-10 dataset. CIFAR-10 contains 60,000 color images of size 32 × 32 pixels across 10 categories, with 50,000 images used for training and 10,000 for testing. To evaluate the robustness of the detection method under different attack strengths, four sets of adversarial examples with varying perturbation upper bounds (*ε* ∈ {0.05, 0.1, 0.2, 0.3}) were constructed.

The detailed dataset partitioning is presented in Table 2. Clean samples are employed as the baseline, while adversarial samples are divided into training and testing sets according to different *ε* values. This ensures that the proposed detection method can be comprehensively evaluated across various levels of attack strength.

**Figure 7:**
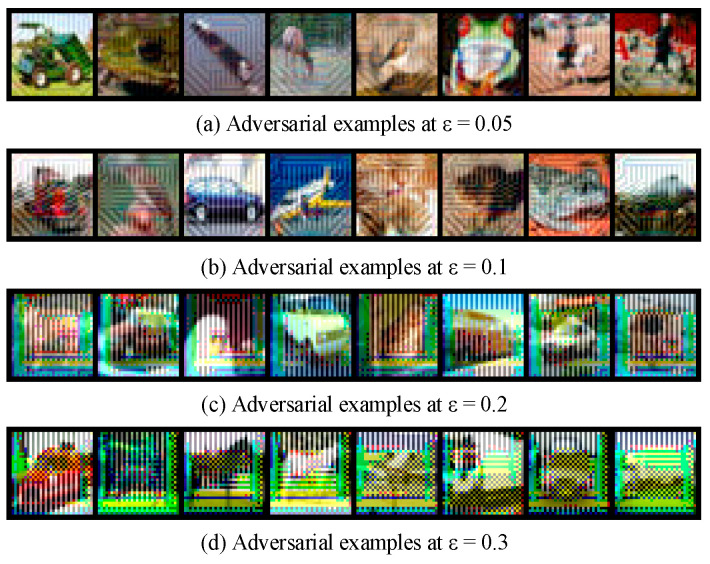
Adversarial sample images.

To provide an intuitive demonstration of the visual characteristics of adversarial samples under different attack strengths, Figure 7 illustrates four groups of adversarial examples. As shown in Figure 7, adversarial examples under varying *ε* values demonstrate perturbations that are imperceptible at low intensities (such as *ε* = 0.05), mimicking stealthy attacks on IoT vision systems where attackers often minimize visual changes to evade detection. This aligns with IoT security needs, as such perturbations could compromise real-time decision-making in applications like autonomous drones or smart surveillance.

Through this systematic dataset construction and visual analysis, a comprehensive data foundation and intuitive visual reference are provided for the subsequent adversarial example detection experiments, thereby facilitating an in-depth evaluation of the detection method’s performance under varying attack intensities. Moreover, such a design is particularly relevant for IoT vision systems, where adversarial perturbations of different strengths may arise in practical deployments.

### 4.2. Comparative Experiments on Different Models

This section presents a detailed comparison and critical analysis of the proposed unsupervised detection framework against existing state-of-the-art methods under various attack intensities. To ensure a comprehensive understanding, the analysis is divided into two parts: (1) performance analysis based on accuracy metrics (AUROC, AUPR) and (2) efficiency analysis evaluating inference time and memory consumption. Thus, six types of detection approaches were compared on the CIFAR-10 dataset:

(1) Deep kNN [16], a nearest-neighbor-based detection approach leveraging deep feature representations;
(2) U-CAN [15], a recently proposed universal adversarial detection framework;
(3) FS [17], which detects adversarial samples by comparing model predictions before and after input quantization and smoothing;
(4) MagNet [18], a defense mechanism that reconstructs inputs using autoencoders to identify abnormal samples;
(5) LiBRe [19], a lightweight Bayesian refinement-based detection approach utilizing prediction uncertainty; and
(6) The proposed A + B method, which integrates spatial feature statistics (Module A) and multidimensional statistical characteristics (Module B) for unsupervised adversarial example detection.

Both ResNet50 and VGG16 were adopted as backbone networks to verify the generality of the detection methods. The evaluation metrics include AUROC and AUPR, measured across a wide range of perturbation strengths, from slight perturbations (*ε* = 0.05) to severe ones (*ε* = 0.3). The detailed experimental results are summarized in Table 3.

**Figure 8:**
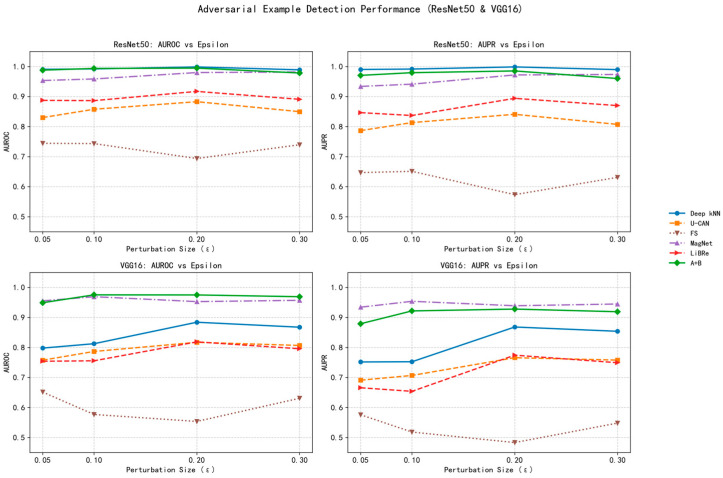
Visualization of model results.

For a more intuitive presentation of the performance comparison trends under different attack intensities, the results above are visualized in a line chart in Figure 8.

From Table 3 and Figure 8, the proposed A + B framework achieves consistently superior detection performance across all attack intensities and architectures. On ResNet50, it attains a peak AUROC of 0.9937 and AUPR of 0.9796 at *ε* = 0.1, while maintaining >0.97 AUPR even under heavy perturbations (*ε* = 0.3). On VGG16, it yields stable high accuracy (AUROC ≈ 0.97 across *ε*), notably surpassing other unsupervised methods such as U-CAN and Deep kNN by >0.15 AUROC on average.

This consistently high performance stems from the complementary theoretical mechanisms of the two modules: (1) Module A captures semantic consistency by comparing each sample to class-level prototypes in spatial statistical space, effectively modeling intra-class coherence. This allows it to detect large semantic shifts when perturbations cause features to deviate from their natural prototypes. (2) Module B, grounded in multivariate statistical theory, detects global distributional anomalies via Mahalanobis distance. It captures subtle, high-order feature inconsistencies invisible to purely spatial methods.

By integrating these two perspectives, the fused detector combines local semantic fidelity with global statistical sensitivity, forming a balanced and attack-agnostic decision boundary. This fusion mitigates the limitations of single-mechanism methods, such as Deep kNN’s over-reliance on neighborhood quality and U-CAN’s dependence on model confidence, and enables generalization across architectures and attack intensities.

Another key observation is that traditional confidence-based methods (U-CAN, FS, and MagNet) exhibit strong sensitivity to perturbation strength, performing reasonably well at small *ε* but degrading sharply as *ε* increases. In contrast, our method maintains stable AUROC even when ε quadruples (from 0.05 to 0.2), evidencing its distributional robustness. This resilience arises because prototype-based and Mahalanobis-based modeling operate directly on internal feature representations rather than prediction logits, making them less vulnerable to confidence manipulation by AdvGAN-generated perturbations.

Moreover, the A + B framework demonstrates stronger cross-architecture robustness: while Deep kNN performs well only on deeper networks (ResNet50) due to high-quality latent manifolds, our approach maintains high accuracy even on VGG16, whose features are less discriminative. This shows that the method effectively compensates for weaker representation quality—an essential property for lightweight IoT devices using shallower CNNs.

Beyond accuracy, computational efficiency is crucial for IoT deployment. Table 4 compares inference time and memory usage across detectors, evaluated on an NVIDIA GeForce GTX 1650 GPU with a batch size of 64. For LiBRe, the Monte Carlo sampling count was *T* = 20.

The proposed method achieves a favorable trade-off between detection strength and computational cost. For ResNet50, the average inference time is only 2.17 ms per image, compared with 5.43 ms for Deep kNN and 28.01 ms for LiBRe. Memory consumption (348 MB) is also moderate, far below LiBRe’s 415 MB despite offering >10% higher AUROC. This efficiency advantage arises from the architecture’s design:

(1) Single forward-pass operation. Both modules reuse intermediate features from the classifier without extra network components or backpropagation, unlike U-CAN and MagNet, which require auxiliary networks or reconstruction paths.
(2) Compact statistical representations. Module A stores only per-class prototypes (≈10 KB for CIFAR-10), while Module B models a low-dimensional covariance matrix of statistical features, avoiding the O(nd) memory explosion of nearest-neighbor methods.
(3) Linear-time fusion. The final score fusion involves only weighted summation of two normalized scores, adding negligible overhead.

This lightweight, training-free design achieves near real-time detection while maintaining high accuracy, aligning well with the computational constraints of embedded IoT hardware such as smart cameras and edge gateways. In comparison, Deep kNN’s O(nd) inference complexity and LiBRe’s heavy Bayesian sampling render them less practical for real-time applications.

In summary, the comparative experiments confirm that the proposed A + B detector achieves state-of-the-art adversarial detection accuracy and efficiency by integrating statistically complementary mechanisms within a unified unsupervised framework. This design not only advances the theoretical foundation of generative attack detection but also offers a practically deployable solution for real-world IoT security applications.

### 4.3. Ablation Study of Module A and Module B

**Figure 9:**
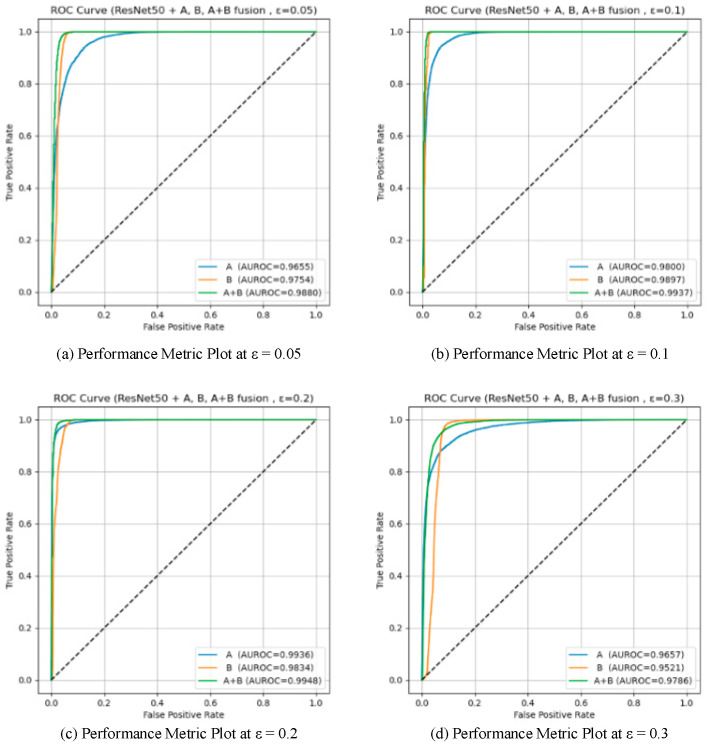
ROC curves for the ablation study on ResNet50.

**Figure 10:**
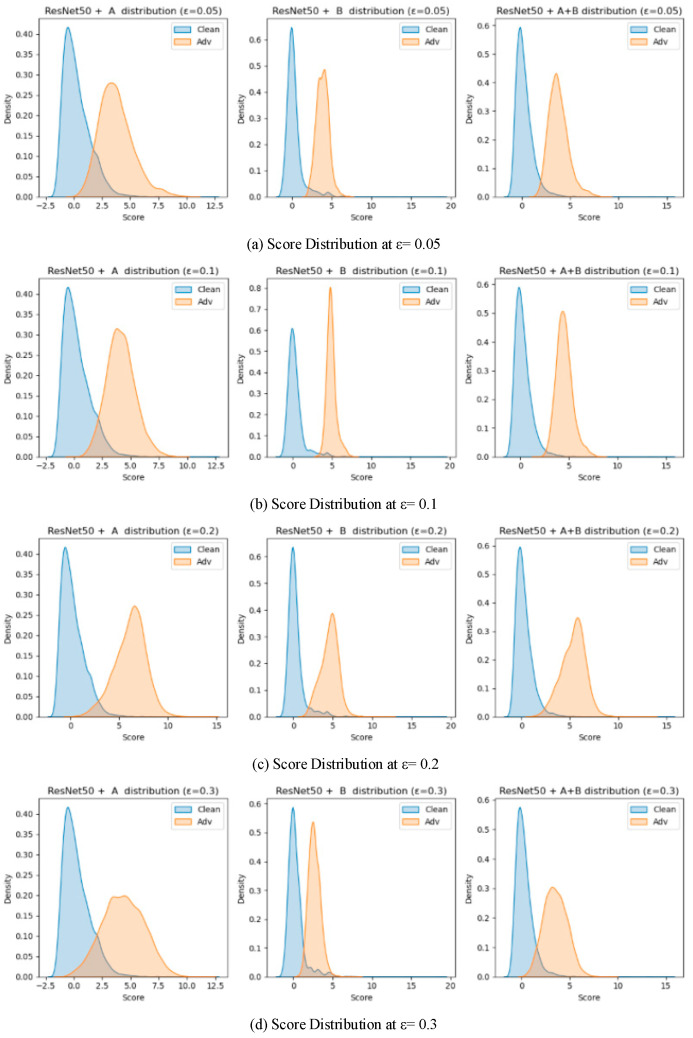
Score distribution plots for the ablation study on ResNet50.

**Figure 11:**
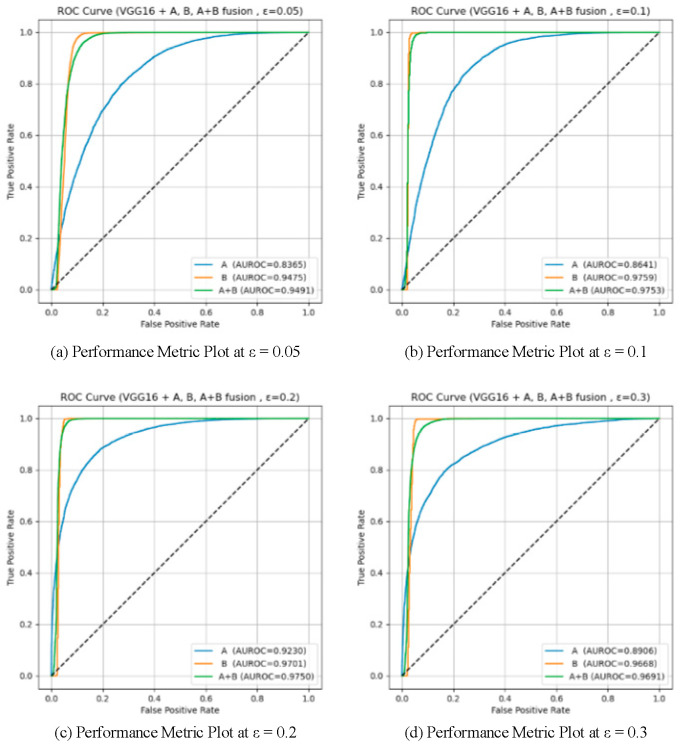
ROC curves for the ablation study on VGG16.

**Figure 12:**
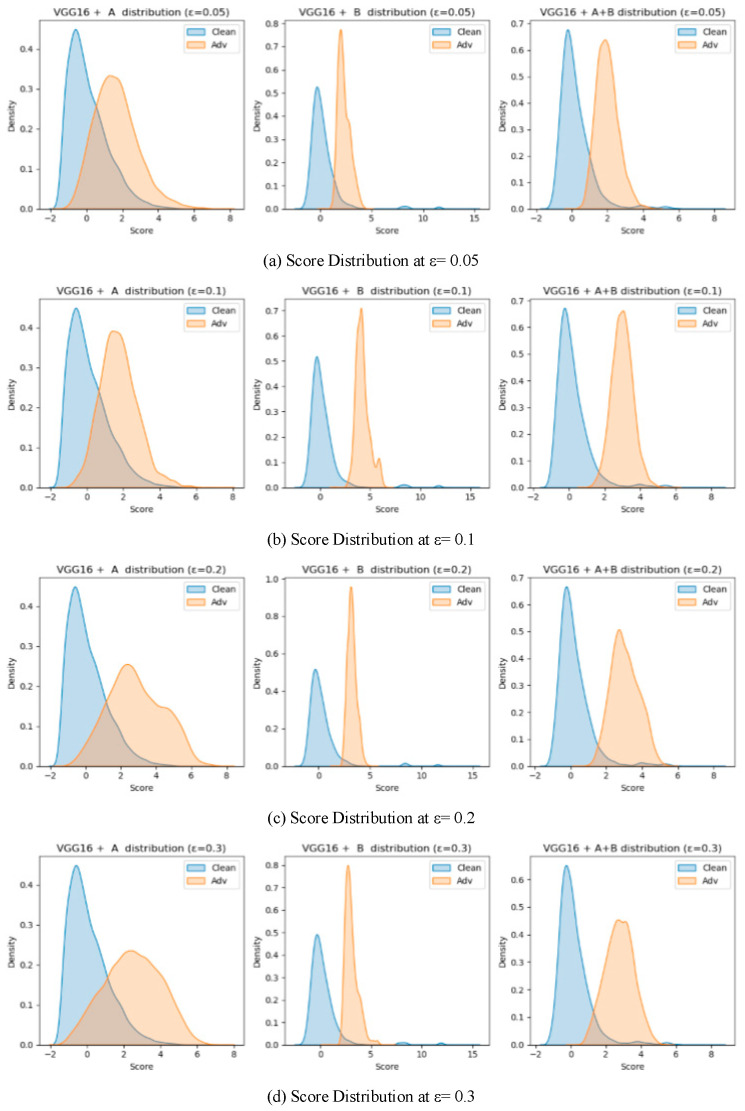
Score distribution plots for the ablation study on VGG16.

To validate the effectiveness of the two proposed detection modules (Module A: unsupervised detection based on spatial prototype similarity, and Module B: distributional anomaly detection combining multidimensional features with Mahalanobis distance), a systematic ablation study was conducted on two baseline models, VGG16 and ResNet50. Four different attack intensities (*ε* ∈ {0.05, 0.1, 0.2, 0.3}) were tested, and three configurations were compared: using only Module A, only Module B, and the fused Module A + B. AUROC and AUPR were used as evaluation metrics. The results are summarized in Table 5. And the results are illustrated in Figure 9, Figure 10, Figure 11 and Figure 12, showing the ROC curves and score distributions for each module and their fusion. In these ROC curves (Figure 9 and Figure 11), the dashed line indicates the performance baseline of a random classifier (AUC = 0.5).

The results obtained on ResNet50 highlight the complementary nature of the two modules.

Module B exhibited higher sensitivity to subtle distributional deviations at low perturbation magnitudes, while Module A showed stronger discriminative capability under larger perturbations. At *ε* = 0.05, Module B achieved a higher AUROC (0.9754) than Module A (0.9655), confirming that Mahalanobis-based global statistics are more effective for capturing fine-grained feature irregularities. However, Module A produced a higher AUPR (0.9527 vs. 0.9357), indicating better stability in precision–recall balance.

As *ε* increased to 0.1, both modules improved, with Module B maintaining AUROC = 0.9897 and Module A reaching 0.9800. The distinction between adversarial and clean feature distributions became more evident, revealing that Module B remained highly sensitive to global statistical shifts while Module A reinforced intra-class consistency detection through spatial aggregation. At *ε* = 0.2, Module A achieved peak performance (AUROC = 0.9936, AUPR = 0.9909), outperforming Module B (0.9834). Under strong perturbation (*ε* = 0.3), Module B suffered a sharp decline (AUROC = 0.9521), whereas Module A retained stable performance (AUROC = 0.9657), illustrating its resilience against large-scale semantic distortions.

These observations can be interpreted through the distinct statistical mechanisms of the two modules. Module B models second- and higher-order dependencies among features, enabling early detection of micro-level distributional anomalies when perturbations remain small. Module A, in contrast, measures prototype-based spatial coherence—a first-order statistic—thereby excelling once perturbations distort global class geometry. The fusion of these complementary cues ensures that both subtle and pronounced deviations are captured effectively. Consequently, the fused detector achieved consistently superior results (AUROC = 0.9937, AUPR = 0.9796 at *ε* = 0.1; AUROC = 0.9786, AUPR = 0.9602 at *ε* = 0.3), confirming the generalization and robustness of the integration strategy.

From a geometric perspective, adversarial perturbations alter the local curvature of the feature manifold. Module B is highly sensitive to these curvature variations near decision boundaries, while Module A responds to broader manifold displacement. Their combination therefore forms a layered defense that remains effective across a wide perturbation range.

On VGG16, the contrast between the two modules was more pronounced due to the shallower architecture and weaker feature separability. At *ε* = 0.05, Module B achieved AUROC = 0.9475, considerably higher than Module A (0.8365), confirming that high-order statistical modeling is more suited to shallow representations where semantic prototypes are less stable. As *ε* increased to 0.1 and 0.2, both modules improved, with Module A gradually narrowing the gap (AUROC = 0.8641 and 0.9230, respectively). At *ε* = 0.3, Module B retained moderate accuracy (AUROC = 0.9668, AUPR = 0.8982) but exhibited larger score overlap, while Module A declined slightly (AUROC = 0.8906) yet maintained better separation stability.

The fusion of the two modules consistently surpassed either single component across all perturbation magnitudes. By integrating Module B’s sensitivity to low-level deviations with Module A’s semantic stability, the fused framework achieved balanced detection capacity across weak and strong perturbation regimes. This complementarity is particularly beneficial for shallow networks and lightweight IoT models, where internal features are less discriminative and noise conditions vary dynamically.

The ablation analysis demonstrates that the proposed integration leverages the statistical complementarity between the two modules: Module B captures covariance-based irregularities indicative of early-stage adversarial perturbations, whereas Module A identifies mean-shift distortions reflecting semantic displacement. Their joint use effectively mitigates the weaknesses of each component—reducing false alarms under low *ε* and preventing missed detections under high *ε*. In practical IoT deployments, such adaptability ensures robustness to both sensor noise and deliberate adversarial interference, providing a computationally efficient and generalizable defense mechanism.

### 4.4. Ablation Study on Layer Selection

The selection of feature layers plays a critical role in the performance stability of adversarial example detection. To systematically evaluate this impact, we conducted comprehensive ablation experiments examining different layer configurations for both ResNet50 and VGG16 architectures across varying perturbation intensities (*ε* = 0.05 to 0.3).

As clearly demonstrated in Table 6, the multi-layer fusion strategy consistently achieves superior performance compared to single-layer configurations. For ResNet50, the combined use of layer3 and layer4 features yields AUROC values ranging from 0.9884 to 0.9948 across different *ε* values, significantly outperforming the individual layer performances. Similarly, for VGG16, the fusion of conv4_3 and conv5_3 features produces AUROC values between 0.9608 and 0.9800, demonstrating substantial improvements over single-layer configurations.

**Figure 13:**
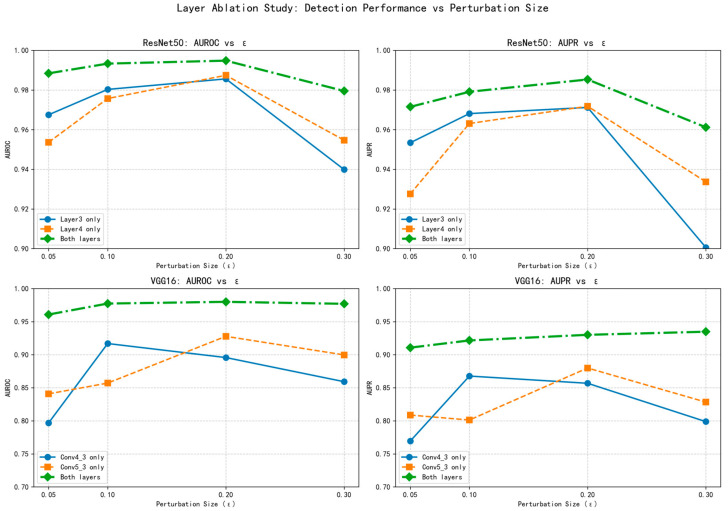
Visualization of layer ablation study.

The visualization in Figure 13 provides further insights into the performance stability across different perturbation intensities. The multi-layer approach maintains consistently high detection performance regardless of the attack strength, while single-layer configurations exhibit greater performance fluctuations. This stability is particularly evident under strong perturbations (*ε* = 0.3), where the fusion strategy demonstrates remarkable robustness compared to the more variable performance of individual layers.

The complementary nature of different layer features contributes significantly to this stability. Shallower layers (conv4_3 in VGG16, layer3 in ResNet50) capture detailed spatial information that is sensitive to subtle perturbations, while deeper layers (conv5_3 in VGG16, layer4 in ResNet50) encode semantic representations that are more robust to larger distortions. The fusion of these complementary characteristics creates a more comprehensive detection mechanism that adapts effectively to varying attack strengths.

Notably, the performance advantage of multi-layer fusion is consistent across both network architectures, though the magnitude of improvement differs. ResNet50 benefits from its inherent residual connections that facilitate feature reuse, while VGG16 shows more pronounced improvements from layer fusion, likely due to its simpler sequential architecture. This cross-architectural consistency underscores the general applicability of the proposed multi-layer fusion approach.

The experimental results confirm that strategic layer selection is crucial for maintaining detection stability against adversarial examples of varying intensities. The multi-layer fusion approach not only enhances detection accuracy but also ensures consistent performance across different attack scenarios, making it particularly valuable for practical applications where attack parameters may be unknown or variable.

### 4.5. Experiments on Traditional Attacks

To further validate the generalizability of the proposed method and to ensure that its effectiveness is not limited to generative adversarial attacks (AdvGAN), this study introduced three common traditional attacks on the CIFAR-10 dataset: Fast Gradient Sign Method (FGSM), Projected Gradient Descent (PGD), and Carlini & Wagner (C&W) attack. The experiments were conducted on two baseline models, VGG16 and ResNet50. The perturbation magnitude was set to *ε* ∈ {0.05, 0.1, 0.2} (note that the C&W attack does not rely on the *ε* parameter). The evaluation metrics were consistent with those described previously, using AUROC and AUPR to assess detection performance.

As shown in Table 7, the proposed method maintained high detection performance under traditional attack scenarios. On the VGG16 model, the AUROC for detecting FGSM attacks remained around 0.95 across different perturbation magnitudes. Under PGD attacks, both AUROC and AUPR were close to 1.0, indicating strong robustness against iterative attacks. The C&W attack posed a greater detection challenge due to its distinct optimization objective and generation mechanism; nevertheless, our method still achieved AUROC = 0.7995 and AUPR = 0.7407 on VGG16. For the ResNet50 model, the detection performance was even more remarkable: AUROC and AUPR approached 0.99 for FGSM and PGD attacks. In the case of C&W, the method achieved AUROC = 0.8571 and AUPR = 0.7789, which is significantly higher than random detection levels.

The above results demonstrate that the proposed method is not only effective against generative attacks such as AdvGAN, but also exhibits strong detection capability under traditional attacks including FGSM, PGD, and C&W. This verifies both the generalizability and robustness of the method, providing a solid foundation for its application in broader adversarial threat scenarios. In particular, the consistent results across different models highlight its potential for enhancing the security of IoT vision systems, where models are often resource-constrained yet exposed to a wide variety of adversarial attacks.

### 4.6. Cross-Dataset Generalization Experiments

To comprehensively evaluate the generalization capability of the proposed method across diverse datasets, CIFAR-10 and Fashion-MNIST datasets were used. These experiments aimed to assess whether the detection framework maintains robust performance when applied to data domains with different characteristics, such as color images (CIFAR-10) and grayscale clothing images (Fashion-MNIST). This evaluation is particularly relevant for IoT security applications, where deployed models may encounter varied visual data from different sensors and environments.

The experiments were performed under four attack intensities (*ε* ∈ {0.05, 0.1, 0.2, 0.3}) using AdvGAN-generated adversarial examples. For Fashion-MNIST, images were resized to 32 × 32 pixels and converted to 3-channel format to ensure compatibility with the VGG16 and ResNet50 models trained on CIFAR-10. The results, measured by AUROC and AUPR metrics, are systematically presented in Table 8.

The results demonstrate outstanding cross-dataset generalization performance. For ResNet50, the method achieved remarkably consistent results across both datasets, with Fashion-MNIST showing slightly superior performance at higher attack intensities (for example, at *ε* = 0.2, AUROC = 0.9954 on Fashion-MNIST vs. 0.9948 on CIFAR-10). This suggests that the residual connections and deeper feature extraction capabilities of ResNet50 provide robust feature representations that transfer well across domains, even when the model was originally trained on CIFAR-10.

For VGG16, more significant performance improvements are observed on Fashion-MNIST compared to CIFAR-10, particularly in AUPR metrics (for example, at *ε* = 0.1, AUPR = 0.9648 on Fashion-MNIST vs. 0.9218 on CIFAR-10). This indicates that the proposed method’s reliance on spatial statistics and multidimensional features is particularly effective for the cleaner, more structured patterns in fashion images, where clothing items exhibit stronger intra-class consistency and inter-class discriminability compared to the more varied natural objects in CIFAR-10.

Notably, the method maintained high performance (AUROC > 0.95 and AUPR > 0.9) across all tested conditions, including the most challenging high-perturbation scenarios (*ε* = 0.3). This consistency underscores the robustness of the feature extraction and anomaly detection approach, which appears less sensitive to dataset-specific characteristics than to the fundamental distributional shifts caused by adversarial perturbations.

These findings have important implications for IoT security applications, where models must often operate on diverse visual data from different sources and environments. The demonstrated generalization capability suggests that the proposed method can be deployed in various IoT vision systems without requiring retraining or adaptation, providing a practical solution for real-world adversarial detection where attack patterns may vary across devices and scenarios. While CIFAR-10 and Fashion-MNIST serve as effective benchmarks for initial validation, future work will evaluate the method on authentic IoT datasets (for example, video feeds from traffic cameras or sensor streams from industrial IoT platforms) to further solidify its practicality in resource-constrained environments.

## 5. Conclusions

The core contribution of this study lies in the proposal of an unsupervised detection framework targeting generative adversarial attacks, which demonstrates significant advantages in terms of practicality and specificity compared with traditional methods. Compared with prior unsupervised detectors relying on confidence or local feature neighborhoods, the proposed dual-module fusion explicitly models both spatial and statistical abnormalities, yielding superior robustness against generative attacks while maintaining lightweight, training-free operation suitable for IoT devices. First, unlike supervised detection models that require a large number of known adversarial samples for training, the proposed method constructs a detector using only clean samples, thereby greatly reducing reliance on prior attack knowledge. This property makes it particularly suitable for real-world IoT security scenarios, where novel and unknown attacks frequently emerge. Second, whereas most existing detection studies focus on optimization-based attacks such as FGSM and PGD, this work systematically investigates detection strategies specifically designed for generative attacks such as AdvGAN. Experimental results demonstrate that by integrating spatial statistics and multidimensional feature abnormalities, the proposed method can effectively capture the unique distributional shifts induced by generative attacks, providing a novel technical perspective for defending against such threats.

Nevertheless, this study still has certain limitations. Most of the experiments were performed in a controlled laboratory environment, leaving open questions about the feasibility, efficiency, and robustness of the framework in real-world IoT deployments.

To address these limitations, future work will proceed in the following directions: (1) extending the experiments to larger-scale and more complex datasets (such as CIFAR-100 [14] and ImageNet [28]) to evaluate the generalization ability of the method; (2) incorporating a broader range of generative adversarial attacks (such as those based on StyleGAN [29]) to enhance the applicability of the detection framework; and (3) deploying the framework in real-world IoT environments to validate its effectiveness and robustness under practical operational conditions.

## Figures and Tables

**Table 1:** Comparison of recent state-of-the-art adversarial detection methods and the proposed framework.

Method	Year	Supervision	Key Principle	Limitation
MagNet [18]	2017	No adversarial labels but requires reconstruction network	Autoencoder-based reconstruction error detection and projection correction	Requires training additional networks; limited generalization to advanced attacks
FS [17]	2018	Heuristic/threshold-based	Input squeezing and prediction consistency check	Weak robustness against complex or generative attacks; susceptible to adaptive attacks
LiBRe [19]	2021	Unsupervised	Bayesian uncertainty and probabilistic inference for anomaly detection	Computationally expensive due to sampling; weak spatial interpretability
Deep kNN [16]	2022	Unsupervised	Layer-wise neighbor consistency in deep feature space	High memory and retrieval cost; performance depends on feature quality
U-CAN [15]	2025	Unsupervised (contrastive/self-supervised)	Contrastive auxiliary networks to learn separable activation patterns	Sensitive to training strategy and sampling; robustness to GAN attacks requires further evaluation
Proposed (A + B)	2025	Unsupervised	Spatial prototypes + Mahalanobis multidimensional statistics	Lightweight and robust without adversarial samples; future work will explore larger IoT datasets

**Table 2:** Configuration of experimental environment and model parameters.

Data Type	*ε* = 0 (Clean Samples)	*ε* = 0.05	*ε* = 0.1	*ε* = 0.2	*ε* = 0.3
Training Set Path	cifar10/train	cifar10_adv(0.05)/train	cifar10_adv(0.1) /train	cifar10_adv(0.2) /train	cifar10_adv(0.3) /train
Test Set Path	cifar10/test	cifar10_adv(0.05)/test	cifar10_adv(0.1) /test	cifar10_adv(0.2) /test	cifar10_adv(0.3) /test

**Table 3:** Comparative results of different models.

*ε*	Backbone	Model	AUROC	AUPR
0.05	ResNet50	Deep kNN	0.9908	0.9903
		U-CAN	0.8301	0.7867
		FS	0.7445	0.6469
		MagNet	0.9535	0.9339
		LiBRe	0.8874	0.8465
		A + B	0.9880	0.9707
	VGG16	Deep kNN	0.7980	0.7519
		U-CAN	0.7577	0.6911
		FS	0.6516	0.5761
		MagNet	0.9553	0.9342
		LiBRe	0.7542	0.6659
		A + B	0.9491	0.8791
0.1	ResNet50	Deep kNN	0.9922	0.9917
		U-CAN	0.8578	0.8134
		FS	0.7434	0.6511
		MagNet	0.9587	0.9414
		LiBRe	0.8865	0.8370
		A + B	0.9937	0.9796
	VGG16	Deep kNN	0.8126	0.7525
		U-CAN	0.7868	0.7069
		FS	0.5769	0.5184
		MagNet	0.9691	0.9538
		LiBRe	0.7557	0.6540
		A + B	0.9753	0.9218
0.2	ResNet50	Deep kNN	0.9987	0.9988
		U-CAN	0.8830	0.8410
		FS	0.6938	0.5734
		MagNet	0.9800	0.9721
		LiBRe	0.9172	0.8941
		A + B	0.9948	0.9854
	VGG16	Deep kNN	0.8841	0.8682
		U-CAN	0.8173	0.7660
		FS	0.5537	0.4834
		MagNet	0.9528	0.9390
		LiBRe	0.8187	0.7744
		A + B	0.9750	0.9279
0.3	ResNet50	Deep kNN	0.9891	0.9898
		U-CAN	0.8498	0.8074
		FS	0.7396	0.6310
		MagNet	0.9820	0.9736
		LiBRe	0.8909	0.8700
		A + B	0.9786	0.9602
	VGG16	Deep kNN	0.8676	0.8540
		U-CAN	0.8068	0.7580
		FS	0.6309	0.5482
		MagNet	0.9571	0.9448
		LiBRe	0.7958	0.7493
		A + B	0.9691	0.9191

**Table 4:** Efficiency of Different Detection Methods: Inference Time vs. Memory Usage.

Model	Backbone	Training Required?	Inference AvgTime (ms/img)	Memory Usage (MB)
U-CAN	VGG16	Yes	0.47	637.32
	ResNet50		1.41	338.15
Deep kNN	VGG16	No	2.12	626.65
	ResNet50		5.43	322.85
FS	VGG16	No	2.22	628.15
	ResNet50		4.88	324.36
MagNet	VGG16	Yes	0.45	544.08
	ResNet50		0.46	121.34
LiBRe	VGG16	No	9.13	1139.89
	ResNet50		28.01	415.61
A + B	VGG16	No	1.25	629.66
	ResNet50		2.17	348.38

**Table 5:** Results of the ablation study.

*ε*	Backbone	Module Setting	AUROC	AUPR
0.05	ResNet50	+A	0.9655	0.9527
		+B	0.9754	0.9357
		+A+B	0.9880	0.9707
	VGG16	+A	0.8365	0.8010
		+B	0.9475	0.8664
		+A+B	0.9491	0.8791
0.1	ResNet50	+A	0.9800	0.9677
		+B	0.9897	0.9664
		+A+B	0.9937	0.9796
	VGG16	+A	0.8641	0.8161
		+B	0.9759	0.9141
		+A+B	0.9753	0.9218
0.2	ResNet50	+A	0.9936	0.9909
		+B	0.9834	0.9603
		+A+B	0.9948	0.9854
	VGG16	+A	0.9230	0.9191
		+B	0.9701	0.9021
		+A+B	0.9750	0.9279
0.3	ResNet50	+A	0.9657	0.9612
		+B	0.9521	0.8803
		+A+B	0.9786	0.9602
	VGG16	+A	0.8906	0.8901
		+B	0.9668	0.8982
		+A+B	0.9691	0.9191

**Table 6:** Results of the layer configuration ablation study.

*ε*	Backbone	Layer Configs	AUROC	AUPR
0.05	ResNet50	Layer3 only	0.9675	0.9534
		Layer4 only	0.9536	0.9276
		Both layers	0.9884	0.9715
	VGG16	Conv4_3 only	0.7968	0.7694
		Conv5_3 only	0.8408	0.8087
		Both layers	0.9608	0.9107
0.1	ResNet50	Layer3 only	0.9803	0.9681
		Layer4 only	0.9757	0.9631
		Both layers	0.9933	0.9791
	VGG16	Conv4_3 only	0.9169	0.8677
		Conv5_3 only	0.8571	0.8012
		Both layers	0.9774	0.9216
0.2	ResNet50	Layer3 only	0.9856	0.9712
		Layer4 only	0.9874	0.9718
		Both layers	0.9948	0.9853
	VGG16	Conv4_3 only	0.8957	0.8568
		Conv5_3 only	0.9278	0.8799
		Both layers	0.9800	0.9301
0.3	ResNet50	Layer3 only	0.9399	0.9006
		Layer4 only	0.9547	0.9337
		Both layers	0.9795	0.9612
	VGG16	Conv4_3 only	0.8592	0.7988
		Conv5_3 only	0.8997	0.8283
		Both layers	0.9770	0.9348

**Table 7:** Detection results against different types of traditional attacks (CIFAR-10 dataset).

Backbone	Attack	*ε*	Attack Success Rate	AUROC	AUPR
VGG16	FGSM	0.05	0.9270	0.9599	0.9538
	FGSM	0.10	0.9110	0.9559	0.9459
	FGSM	0.20	0.8910	0.9747	0.9723
	PGD	0.05	0.9980	0.9912	0.9889
	PGD	0.10	0.9979	0.9910	0.9990
	PGD	0.20	0.9934	0.9935	0.9876
	CW	—	1.0000	0.7995	0.7407
ResNet50	FGSM	0.05	0.8700	0.9159	0.8725
	FGSM	0.10	0.8790	0.9892	0.9719
	FGSM	0.20	0.8790	0.9974	0.9931
	PGD	0.05	1.0000	0.9988	0.9985
	PGD	0.10	0.9989	0.9999	0.9999
	PGD	0.20	1.0000	0.9987	0.9983
	CW	—	0.9990	0.8571	0.7789

**Table 8:** Cross-dataset detection results for AdvGAN attacks.

Model	Dataset	*ε*	AUROC	AUPR
ResNet50 + A + B	CIFAR-10	0.05	0.9880	0.9707
		0.1	0.9937	0.9796
		0.2	0.9948	0.9854
		0.3	0.9786	0.9602
	Fashion-MNIST	0.05	0.9901	0.9864
		0.1	0.9929	0.9898
		0.2	0.9954	0.9912
		0.3	0.9876	0.9825
VGG16 + A + B	CIFAR-10	0.05	0.9491	0.8791
		0.1	0.9753	0.9218
		0.2	0.9750	0.9279
		0.3	0.9691	0.9191
	Fashion-MNIST	0.05	0.9522	0.9426
		0.1	0.9719	0.9648
		0.2	0.9760	0.9703
		0.3	0.9711	0.9676

## Data Availability

The original datasets presented in the study are openly available in CIFAR-10 at https://www.cs.toronto.edu/~kriz/cifar.html (accessed on 28 September 2025) and Fashion-MNIST at https://www.worldlink.com.cn/en/osdir/fashion-mnist.html (accessed on 29 September 2025).
